# Supplementary material for: Chirality-controlled crystallization via screw dislocations
Source: Nat Commun. 2018 Apr 11;9:1405. doi: 10.1038/s41467-018-03745-4 (PMC5895742; doi:10.1038/s41467-018-03745-4)
Supplement: Supplementary file 1 — Supplementary Information(PDF 386 kb) [file 41467_2018_3745_MOESM1_ESM.pdf]

Supplementary Figures for

Chirality-controlled crystallization via screw dislocations

B. Sung, A. de la Cotte, and E. Grelet

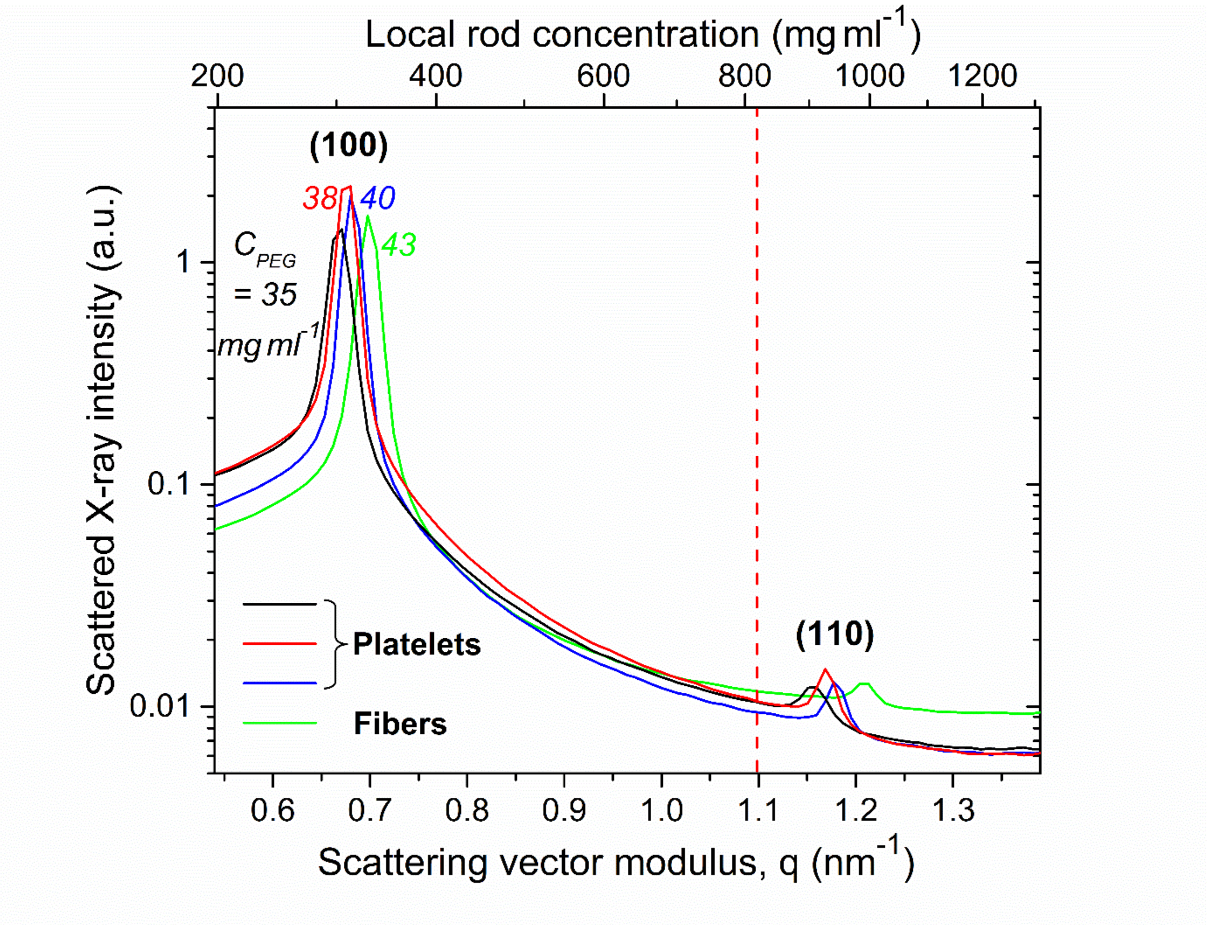

Supplementary Figure 1: Small angle X-ray scattering measurements of hexagonal platelets and columnar fibers of rod-like viruses with varying depleting polymer (PEG) concentration. All the curves correspond to a hexagonal long-ranged positional order with the presence of (100) and (110) Bragg refractions. From the 2D swelling law of the hexagonal ordering of rod-like viruses (See 'Methods'), the local virus concentration in the platelets is estimated to be ~300 mg ml^{-1}. The vertical dotted red line indicates the 2D hexagonal close packing concentration (820 mg ml^{-1}).

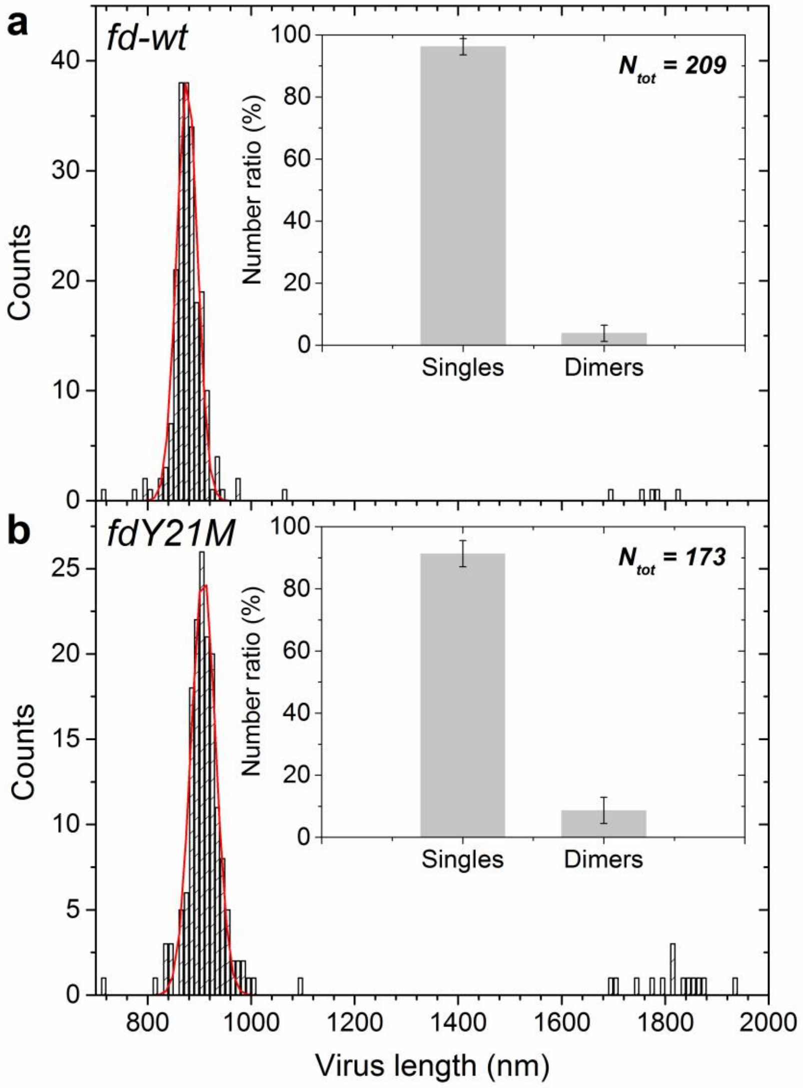

Supplementary Figure 2: Virus contour length distributions measured by transmission electron microscopy. Measurements performed for **a**: fd-wt and **b**: fdY21M virus strains. A fraction of dimers of 4% and 9% is found in fd-wt and fdY21M batches, respectively. The red lines correspond to a Gaussian fits of the main peak. Note that multimeric phages of higher length (trimers, tetramers,…) exist within a fraction too low to be measured from our representative statistical samples, and are therefore present in a fraction lower than 1% in our batches. This estimation and the error bars in the inset have been calculated using a confidence level of 95%.
