# Supplementary material for: Chirality-controlled crystallization via screw dislocations
Source: Nat Commun. 2018 Apr 11;9:1405. doi: 10.1038/s41467-018-03745-4 (PMC5895742; doi:10.1038/s41467-018-03745-4)
Supplement: Supplementary file 3 — Description of Additional Supplementary Files(PDF 62 kb) [file 41467_2018_3745_MOESM3_ESM.pdf]

Description of Additional Supplementary Files

File Name: Supplementary Movie 1
Description: Time-stream DIC/fluorescence overlaid images acquired simultaneously (See 'Methods') of a hexagonal platelet viewed side-on. At the platelet edge, red fluorescent labeled viruses (added with a ratio of $1:10^5$ to non-labeled ones) stand at their lattice position without any detectable (by optical microscopy) self-diffusion along or normal to their long axis. This confirms the long-ranged positional ordering within the platelets, as demonstrated by SAXS (Supplementary Figure 1). Scale bar, 1 µm.

File Name: Supplementary Movie 2
Description: Time-stream DIC/fluorescence overlaid images of a central protruding defect belonging to a hexagonal platelet viewed edge-on. The absence of any detectable virus motion within the defect structure confirms the long-ranged positional order. Scale bar, 1 µm.

File Name: Supplementary Movie 3
Description: Z-stack DIC microscopy images of a platelet (fd-wt strain) exhibiting a left-handed screw dislocation observed with an objective driven by a piezo-focus device. The handedness is determined by following the in-focus edge of the platelet as increasing the z-position. Scale bar, 5 µm.

File Name: Supplementary Movie 4
Description: Z-stack images of a face-on platelet (fd-wt strain) showing a right-handed screw dislocation. The images were obtained by DIC microscopy coupled with a piezo z-positioning objective. Scale bar, 2 µm.

File Name: Supplementary Movie 5
Description: Z-stack images by fluorescence microscopy of the same platelet as the one observed in Supplementary Movie 4. The platelet edges are decorated by fluorescently labeled viral rods. Approximately 1:1000 virus particles were fluorescently labeled with green dyes. Because they chemically slightly differ from bare viruses, most of the labeled viruses cannot incorporate the platelet and are finally assembled at the edges thanks to depletion interaction. Scale bar, 2 µm.

File Name: Supplementary Movie 6
Description: Kinetics of growth of crystalline platelets (DIC video microscopy). **1**, **2** and **3** are viewed from the side: if **1** and **2** are flat and therefore achiral, **3** is twisted and therefore exhibits a screw dislocation. The orientation of **4** changes with time. Note that some focus-out can appear due to the Brownian motion of the platelets. Scale bar, 2 µm.

File Name: Supplementary Movie 7
Description: Kinetic growth of a hexagonal platelets observed by DIC microscopy. The 2D growth starts from an edge-on nucleus which then rotates and continues to grow in a face-on orientation. The hexagonal faceting appears only clearly after some time, suggesting a roughening-like transition. Some focus adjustment has been performed during the experiment. Scale bar, 2 µm.

File Name: Supplementary Movie 8
Description: Time-stream DIC/fluorescence overlaid images of a helical platelet viewed from the side, for which the crystallization by particle attachment of a single labeled virus initially in the liquid suspension is evidenced. After joining the platelet edge, the particle continues to slightly diffuse laterally (normal to its long axis), interpreted as optimizing its position within the hexagonal lattice. Scale bar, 1 µm.
